# Epidemiological features of tuberculosis infection in a high-altitude population: a population-based, cross-sectional survey in Tibet, China

**DOI:** 10.3389/fcimb.2025.1651920

**Published:** 2025-09-02

**Authors:** Jian Wang, Shaojun Pei, Guofeng Yang, Nima Qucuo, Qifei Song, Xiaoqiu Liu, Xue Li, Wei Chen, Tao Li, Eryong Liu, Xichao Ou, Hui Chen, Ni Ni, Jingjuan Ren, Yanlin Zhao, Hongqiang Gong

**Affiliations:** ^1^ Centre for Disease Control and Prevention of Tibetan Autonomous Region, Tibet, Lhasa, China; ^2^ Department of Global Health, School of Public Health, Peking University, Beijing, China; ^3^ Division of General Internal Medicine and Primary Care, Brigham and Women’s Hospital, Boston, MA, United States; ^4^ National Center for Tuberculosis Control and Prevention, Chinese Centre for Disease Control and Prevention (Chinese Academy of Preventive Medicine), Beijing, China; ^5^ National Key Laboratory of Intelligent Tracking and Forecasting for Infectious Diseases, Chinese Center for Disease Control and Prevention (Chinese Academy of Preventive Medicine), Beijing, China

**Keywords:** tuberculosis infection, high altitude, very high altitude, Tibet, ESAT6-CFP10 skin test

## Abstract

**Background:**

Targeted strategies for marginalized populations, including high-altitude communities, are crucial for TB elimination. This study assessed TB infection (TBI) prevalence across altitudinal gradients and evaluated altitude-dependent risk factors in Tibet, China.

**Methods:**

A cross-sectional survey by multistage stratified random cluster sampling was conducted using ESAT6-CFP10 skin test (C-TST), symptom screening, chest X-rays, and bacteriological tests. The influencing factors of C-TST positivity were analyzed via generalized linear mixed models (GLMMs) and Boruta algorithm feature ranking. The TBI prevalence was estimated using WHO-recommended methods. Causal mediation analysis was performed to explore mediating variables contributing to association between altitude and TBI prevalence.

**Results:**

The estimated TBI prevalence in Tibet was 20.7% (95% CI 14.3%-33.0%). Residential altitude was the strongest predictor of C-TST positivity (aOR=0.53, p<0.001). The interaction analyses revealed significant modification effects of both smoking status (interaction p=0.0065) and BCG vaccination (interaction p=0.028) on the altitude-C-TST positivity association. Mediation analysis indicated that the observed inverse relationship between study site altitude and crude TBI prevalence was mediated by per capita land space (IE= -6.29e-05, p=0.04). The prevalence of TBI in very high-altitude (VHA) areas was 12.8%, approximately one-third of that in high-altitude (HA) areas (35.0%). Stratified analyses revealed distinct risk profiles - occupational exposures predominated in HA regions, whereas physiological factors (age, BMI, smoking) drove positivity in VHA areas.

**Conclusion:**

Our results suggest that TB infection is significantly associated with altitude, necessitating accelerated research into plateau-specific disease mechanisms and the development of targeted public health strategies tailored to local socio-medical conditions. This integrated biological and socio-economic approach is essential to overcome the compounded vulnerabilities of high-altitude populations and ensure that China equitably achieves its goal of eliminating TB.

## Background

Despite substantial progress to combat tuberculosis (TB), the infection remains a major global health problem. The global prevalence of tuberculosis infection (TBI) is closer to one-fourth, with large regional differences (Cohen et al., 2019). China is one of the countries with a high burden of TB and TBI, with approximately 350 million persons living with the infection (Gao et al., 2015; Houben and Dodd, 2016). Preventing TBI and stopping progression from infection to disease are critical for reducing TB incidence to the levels envisaged by the End TB Strategy (Uplekar et al., 2015).

It is estimated that over 81.6 million people living at high altitude in the world (Tremblay and Ainslie, 2021). High-altitude regions encompass several countries with high TB burdens, including Peru, Bolivia, India, China, Nepal, and Ethiopia. These areas collectively contribute approximately 8-12% of global TB cases (Gelaw et al., 2019). The synergistic interaction between high-altitude environmental factors and socioeconomic disparities creates a unique epidemiological landscape for tuberculosis transmission. Chronic hypobaric hypoxia, a defining characteristic of these regions, induces evolutionary adaptations such as EPAS1/HIF-2α polymorphisms in Tibetan populations (Tashi et al., 2017). While these genetic adaptations enhance survival in oxygen-deprived environments, they may simultaneously attenuate critical hypoxia-induced inflammatory responses essential for MTB containment (Bucşan et al., 2022). Specifically, these adaptations compromise macrophage antimicrobial functions, potentially facilitating bacterial persistence (Li et al., 2018). These biological vulnerabilities are exacerbated by structural challenges including geographic isolation, socioeconomic barriers to care-seeking, and suboptimal housing conditions that promote airborne transmission (Wang et al., 2023). This multifaceted challenge demands integrated public health strategies that simultaneously address the distinct pathophysiology of high-altitude TB and the systemic healthcare access limitations faced by these populations. Although several epidemiological studies have investigated the association between altitude and tuberculosis incidence, these studies were conducted at relatively early stages, lacked studies in very high altitude (VHA; >3500 m) areas, and seldom focused on tuberculosis infection (Gardiner et al., 1923; Mansoer et al., 1999; Olender et al., 2003; Pérez-Padilla and Franco-Marina, 2004; Vargas et al., 2004; Vree et al., 2007).

Tibet is a provincial autonomous region of China that is located in the Qinghai-Tibet Plateau at an average altitude >4000 m above sea level and ~95% of its population is of Tibetan ethnicity (Wu, 2001). The unique hypobaric hypoxia environment and other related factors may have caused the suboptimal health status among the Tibetan population. Physiological, anthropological and genomic research over the last 50 years has characterized the phenotypic and genotypic differences between Han Chinese and Tibetans living at altitude (Weitz et al., 2016; He et al., 2017). For TB, it has greater burdens than other areas of China. In 2023, the notified incidence of TB in Tibet was 107/100,000 population, ranking third in China (Wang et al., 2024).

To address this evidence gap, the Tibet Autonomous Region CDC implemented a population-based, cross-sectional survey to quantify TBI prevalence across altitudinal gradients and evaluate potential altitude-dependent variations in key epidemiological risk factors.

## Materials and methods

### Study design and participants

Between June 1, 2023, and November 30, 2023, we undertook a population-based, cross-sectional TBI survey of registered residents in the Tibet Autonomous Region of China using a multistage stratified random cluster sampling. We stratified the counties or districts based on TB notified incidence into areas with low (TB notified incidence <77.21 per 100,000 population), medium (TB notified incidence: 77.21-136.71 per 100,000 population), and high incidence (TB notified incidence >136.71 per 100,000 population). We allocated 18 clusters to areas with high incidence, 12 to areas with medium incidence, and 6 to areas with low incidence, which was proportional to the population of each stratum; in addition, it was determined that all cities should have at least one cluster. Within each stratum, the number of clusters assigned to each urban (towns) or rural area (townships) was proportional to urban and rural population size. We defined a cluster as a community in a town or a village in a township. We first selected towns, or townships with simple random sampling. Within the selected towns or townships, we then selected communities or villages with simple random sampling respectively. In each cluster, we visited all households. If the population of eligible individuals in a cluster was fewer than 200 people, then we added part of the next adjoining village or community to reach the target size. If the population of eligible individuals in a cluster was higher than the target size, then randomly selected individuals will be enumerated until the target cluster size is reached.

Inclusion criteria of eligible participants were people aged 5 years or older; household registration or residence permit for that village; continuous residence at the study site for 6 months or longer in the past year; ability to complete the investigations and tests during the study duration; and provision of voluntary written informed consent. Exclusion criteria were registered active tuberculosis, self-reported history of tuberculosis, and pregnancy.

The survey protocol was approved by the Tibet Center for Disease Control and Prevention Ethics Review Committee in May, 2023 (2023-003), with annual renewal until completion. Individual written informed consent or assent and parent or guardian consent for participants younger than 18 years was obtained at survey enrolment. Participation was voluntary, and participants received in-kind reimbursement to the value of US$14 (100 RMB) for time spent on survey activities.

### Procedures

At the screening site, each participant was interviewed with the use of a standard questionnaire, to collect sociodemographic details, comorbidities, and symptoms (Appendix 1). Participants then had recombinant MTB fusion protein ESAT6/CFP10 (C-TST), unless they declined or a C-TST could not be done because of TB under treatment. C-TST (0.1 ml, 5 U) was injected with the medial forearm skin, and the injection site reaction was checked 48–72 h after injection (Xu et al., 2022; Xia et al., 2023). The lengths of the transverse and longitudinal diameters of the redness and sclerosis were measured and recorded, and the larger ones were selected from the red corona and hard nodules, and the average diameter [(transverse diameter + longitudinal diameter)/2] was calculated, of which ≥5 mm was a positive reaction Blistering, necrosis, and lymphangitis were considered strong positive reactions. Digital chest radiography was done in all participants older than 15 years, and in those younger than 15 years with reported suspected symptoms of pulmonary TB (PTB) or history of close contacts. The participants who reported suspected symptoms of PTB or history of close contacts, participants who had an abnormal chest radiograph, participants with a positive C-TST reaction and participants who do not undergo X-ray screening, such as disabled people were requested to submit three sputum samples. The samples will be transported daily to the Tibet Tuberculosis Reference Laboratory under cold chain conditions for GeneXpert and Mycobacterium Growth Indicator Tube (MGIT) liquid culture testing. Participants with symptoms of PTB or with radiographic abnormalities consistent with active pulmonary disease were transferred to the local city level CDC for disease confirmation, in accordance with WHO guidelines.

### Definition of TBI and TB

We defined TB as individuals with a sputum-culture-positive or GeneXpert-positive result, or with suspected pulmonary infection (defined by radiographic abnormalities consistent with active pulmonary infection together with a positive C-TST) (WHO, 2023). TBI was defined as individuals with a positive C-TST and exclusion of TB.

### Definition of HA and VHA

According to the International Society for Mountain Medicine for evaluating the lowered amount of oxygen in the atmosphere of different altitude, we defined high altitude (HA) as 1,500–3,500 m and very high altitude as 3,500–5,500 m (Non-physician altitude tutorial. International society for mountain medicine).

### Statistical analysis

The sample size of 7 200 people with a cluster size of 200 was based on the assumptions of the prevalence of TBI in Tibet estimated at 20%, tolerance error of 20%, design effect of 2&α44, α value of 0.05 and a participation rate of 75%.

Generalized Linear Mixed Effects Model (GLMM) was conducted to explore risk factors of C-TST positivity (Lai et al., 2022). A two-sided p-value of 0.05 or less was regarded as significant. We applied a Boruta algorithm, a random forest (RF) based machine learning approach, to identify relevant predictors of C-TST positivity, with a p-value threshold of 0.01 and a maximal number of 500 runs (Xia et al., 2024). Causal mediation analysis was performed to explore mediating variables contributing to the negative association between altitude and crude TBI prevalence (Imai et al., 2010). Survey prevalence was extrapolated to estimate prevalence of TBI in people aged 5 years old and above and in sub-populations in Tibet using WHO standard methodologies, which accounted for cluster sampling, non-participation, missing data and demographic structure (WHO, 2011; Floyd et al., 2013). The model first imputed multiple missing values for individuals with missing results (the imputed outcome variable was C-TST result, positive or negative). Factors identified as associated with C-TST positivity were incorporated into the imputation model, and 25 imputed datasets were created using the R package Mice (Van Buuren and Groothuis-Oudshoorn, 2011). For each imputed dataset, weights were then calculated based on the total number of individuals and the proportion of individuals sampled within the stratum, living altitude group, age group, and sex. Then inverse probability weighting was used to adjust for differences in survey participation by age, sex, and stratum. Finally, an average of the estimates of pulmonary TB prevalence from each of the imputed datasets was then calculated, together with a 95% confidence interval (CI). Demographic structure data and socio-environmental data of counties (average altitude, and land area per capita) was from China’s seventh national population census (Office of the Leading Group of the State Council for the Seventh National Population Census). The reported cases of PTB were derived from the national Tuberculosis Information Management System (TBIMS). Statistical analyses were done with RStudio version 2023.03.1 + 446.

## Results

**Table 1** shows detailed information about the population assessed. Of 7320 eligible participants, 6533 actually participated including questionnaire and C-TST, with a response rate of 89.3% (**Supplementary Figure S1**). Overall, roughly 55% of participants were female and 12% were 60 years or older in this survey which was higher than the general population of Tibet (Office of the Leading Group of the State Council for the Seventh National Population Census). About 95% are Tibetan. Nearly 60% of participants had a BCG scar. A tenth of participants reported having ever smoked and about a third had ever consumed alcohol. BMI distribution showed that almost a quarter of the participants were overweight or obese.

**Table 1:** Population sampling in the study.

	Stratum 1	Stratum 2	Stratum 3	Total
Number of clusters	18	12	6	36
Notified incidence of active tuberculosis in 2022 (per 100,000 population)	>136.7	77.2~136.7	<77.2	NA
Registered population established by cluster sampling	3719	2467	1234	7420
Excluded because of self-reported tuberculosis on treatment	16	6	2	24
Excluded because of active pulmonary tuberculosis	30	37	9	76
Eligible population included in the baseline survey	3673	2424	1223	7320
Excluded because of absent result from C-TST test	583	111	93	787
Actual population assessed for the crude prevalence of tuberculosis infection	3090	2313	1130	6533

**Figure 1:**
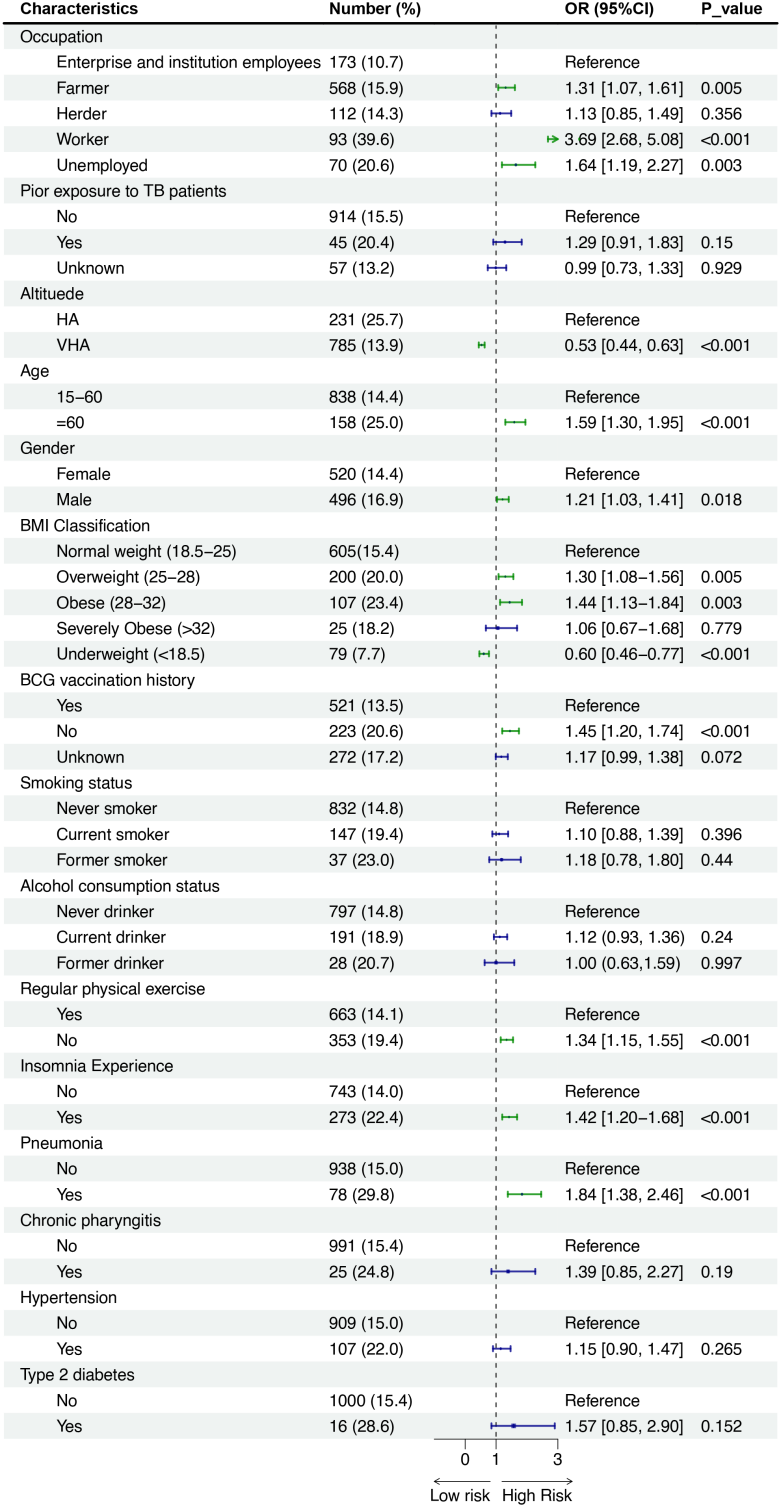
Forest plot showing odds ratios (OR) with 95% confidence intervals (CI) for various characteristics affecting risk. Categories include occupation, prior TB exposure, altitude, age, gender, BMI, BCG vaccination, smoking, alcohol use, exercise, insomnia, pneumonia, chronic pharyngitis, hypertension, and diabetes. Significant risk factors include being a worker, unemployed, certain BMIs, no BCG vaccination, lack of exercise, insomnia, and pneumonia, with P-values indicating statistical significance.

**Figure 2:**
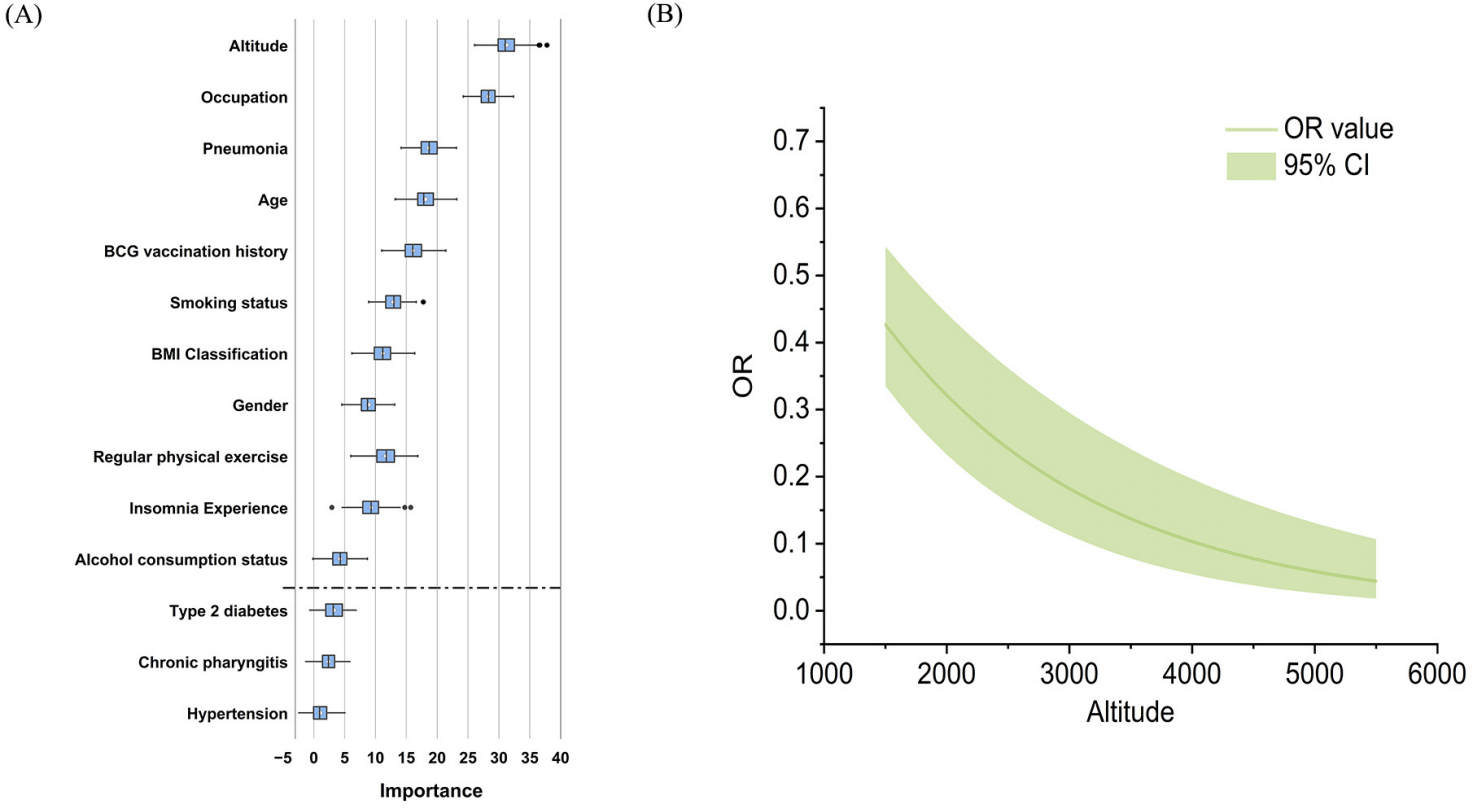
**(A)** Ranked importance of variables identified in Boruta feature selection for C-TST positivity. **(B)** Adjusted OR value curve of the altitude on C-TST positivity.

In total, 1016 (15.5%) of 6533 persons who underwent C-TST had an induration of 5 mm or more, 121 (1.9%) participants had blistering, necrosis, or lymphangitis. In multivariate analysis, the following factors demonstrated significant associations with positive C-TST results: engagement in heavy physical work (farmer and worker), male gender, increasing age, presence of BCG scars, elevated BMI (≥25 kg/m²), residence at high altitude, history of tobacco use, low physical exercise levels, reported insomnia, and previous pneumonia diagnosis (**Figure 1**). The Boruta feature importance analysis identified altitude of residence as the most determinant predictor of C-TST positivity, with an inverse relationship observed - higher altitudes were associated with decreased risk of positive test results (**Figure 2**).

**Figure 3:**
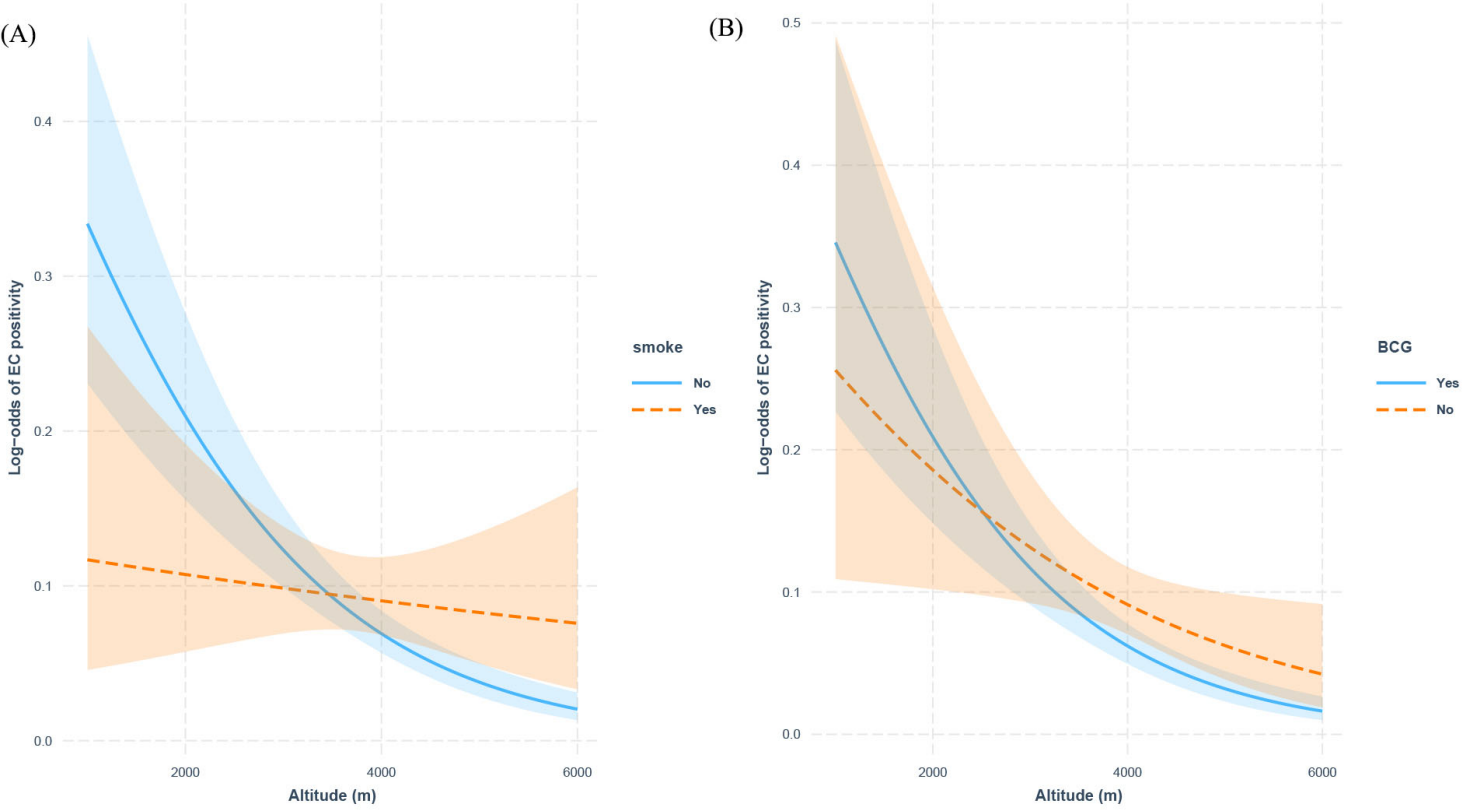
Interaction effects of smoking status and BCG Vaccination on altitude and C-TST positivity: **(A)** smoking status, **(B)** BCG vaccination history.

The interaction analyses revealed significant modification effects of both smoking status (interaction p=0.0065) and BCG vaccination (interaction p=0.028) on the altitude-C-TST positivity association (**Figure 3**). Notably, while each 500-meter elevation increase was associated with a 31.9% reduced C-TST positivity risk among non-smokers (β=-6.37×10^−4^, p<0.001), no significant altitude effect was observed in smokers (β=-9.56×10^−5^, p=0.6). Similarly, BCG-vaccinated individuals demonstrated a stronger inverse altitude association (34.7% risk reduction per 500m, β=-6.93×10^−4^, p<0.001) compared to unvaccinated individuals (17.7% reduction, β=-3.54×10^−4^, p=0.03), representing a 50% attenuation of the altitude protective effect. These findings suggest that both smoking and lack of BCG vaccination may compromise the beneficial physiological adaptations to high-altitude environments.

In subgroup analyses stratified by altitude, distinct epidemiological patterns were observed. Among HA residents, C-TST positivity was significantly associated with occupational exposures, whereas in VHA populations, positivity was predominantly linked to individual physiological factors, including age, sex, BMI, BCG vaccination status and smoking history. Comorbidity profiles also exhibited altitude-dependent variations: in HA regions, pneumonia and type 2 diabetes were significant risk factors for C-TST positivity, whereas in VHA areas, pneumonia, chronic pharyngitis, and hypertension demonstrated stronger associations with positive test results (**Supplementary Figure S2**).

**Figure 4:**
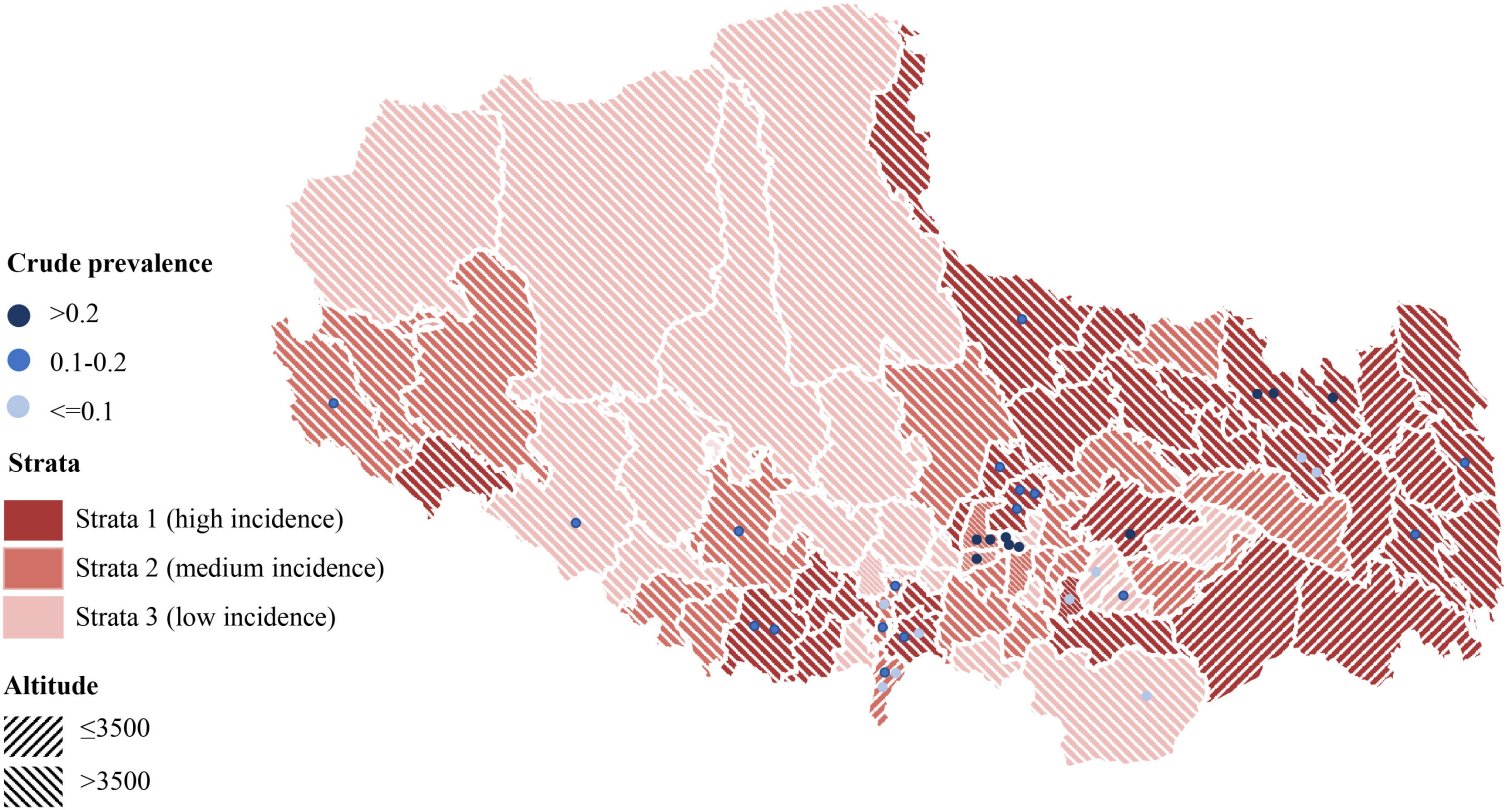
Distribution of clusters tested for TBI and the crude prevalence of sampling sites.

After excluding active TB cases, the crude prevalence of TBI in this survey population was 15.7% (95% CI: 14.7%-16.4%). Geospatial analysis revealed the highest crude prevalence rates in central counties of the province (**Figure 4**). Mediation analysis indicated that the observed inverse relationship between study site altitude and crude TBI prevalence was mediated by per capita land space, accounting for a significant proportion of this association (IE= -6.29e-05, p=0.04, **Supplementary Figure S3**). Using best-practice analytical methods with statistical adjustment, the estimated TBI prevalence among individuals aged 5 years and older in Tibet Autonomous Region was 20.7% (95% CI: 14.3%-33.0%) (**Table 2**). The estimated prevalence of TBI was approximately 1·3 times higher in men than in women, and was highest in people aged 60 years or older. Prevalence was lower in stratum 3 than in strata 2 and 1. The estimated prevalence of TBI in the HA region was nearly 3 times higher than that in the VHA region, but the reported incidence of TB in the HA region was only 1.5 times that in the VHA region (**Table 2**).

**Table 2:** Prevalence of TBI and notified TB incidence in Tibet.

Characteristics	Crude prevalence of TBI (95% CI)	Estimated prevalence of TBI (95% CI)	Notified TB incidence (95% CI) per 100,000 population
Overall	15.7% (14.8%, 16.6%)	20.5% (14.2%, 32.8%)	106.8 (103.5, 110.2)
Sex			
Male	17.1% (15.8%, 18.5%)	22.8% (16.1%, 34.7%)	109.1 (104.5, 113.9)
Female	14.5% (13.4%, 15.7%)	18.1% (12.1%, 30.7%)	104.3 (99.5, 109.2)
Age			
<=15	1.9% (0.9%, 4.1%)	2.1% (0.3%, 21.3%)	23.7 (20.5, 27.3)
16-59	15.2% (14.3%, 16.2%)	23.5% (0.8%, 28.4%)	119.0 (114.7, 123.4)
>=60	25.3% (22.3%, 28.7%)	32.0% (19.8%, 35.2%)	238.1 (221.9, 255.1)
Strata			
1 (low incidence)	16.2% (14.2%, 18.5%)	13.5% (7.5%, 24.7%)	54.8 (51.2, 58.6)
2 (medium incidence)	19.3% (17.8%, 21.0%)	37.5% (29.5%, 48.7%)	101.1 (95.2, 107.2)
3 (high incidence)	12.8% (11.6%, 14.0%)	12.6% (7.8%, 26.5%)	202.6 (193.5, 211.9)
Altitude			
>3500m	14.0% (12.6%, 15.6%)	12.8% (6.9%, 26.7%)	108.3 (104.3, 112.2)
≤3500m	26.0% (24.3%, 27.7%)	35.0% (25.3%, 43.1%)	155.7 (144.8, 167.2)

## Discussion

High-altitude regions face unique public health challenges characterized by extreme environmental conditions, pervasive poverty, limited healthcare access, and low health awareness, all of which exacerbate disease burdens. These areas often experience heightened vulnerability to infectious diseases like TB. Studies are encouraged in countries with considerable high-altitude populations, to understand how the environmental stress (physiological and social) of high altitude impacts physiology, adaptation, health, and disease (Tremblay and Ainslie, 2021). In response, we launched a prospective cohort study in Tibet to investigate MTB infection and disease progression, with this work representing the baseline survey. The results reveal that both the TBI prevalence and TB incidence in the VHA region are lower than those in the HA region, and distinct risk factors exist between the two regions.

Our findings demonstrate a significant inverse association between altitude and TBI risk, with prevalence decreasing as elevation increases. This observation aligns with several previous studies conducted in high-altitude regions. For instance, research in the Peruvian Andes reported a lower TBI prevalence at elevations above 3,000 meters compared to lowland areas (Tashi et al., 2017). Similarly, a study in Turkey found reduced TB infection rates in high-altitude zones (Tanrikulu et al., 2008). However, our findings demonstrate that smoking status and BCG vaccination significantly modify the protective association between altitude and C-TST positivity. The complete attenuation of altitude’s protective effect among smokers suggests that tobacco-induced pulmonary alterations may override the natural protective mechanisms conferred by high-altitude adaptation (Liu et al., 2025). Furthermore, the 50% weaker protective effect observed in BCG-unvaccinated individuals implies that vaccine-induced immunity may synergize with physiological responses to high-altitude hypoxia, potentially enhancing host defense against MTB infection (Zhu et al., 2021). Our mediation and mixed-effects analyses demonstrate that the observed altitude-dependent reduction in TBI risk is likely mediated through two primary pathways: decreased population density at higher elevations (β = -0.22, p=0.02), which reduces transmission opportunities, and altitude-specific physiological adaptations that enhance host defenses. Notably, we found BCG vaccine effectiveness showed significant altitude-dependent enhancement (15% increase per 500m elevation, p=0.015), suggesting high-altitude residents may develop more robust vaccine-induced immunity, potentially through hypoxia-driven immune modulation (HIF-1α upregulation) and vitamin D-mediated pathways (Fabri et al., 2011; Burtscher et al., 2020; Calvo, 2020). These findings provide mechanistic insights into how both environmental and biological factors collectively contribute to the unique TBI epidemiology in high-altitude populations.

Subgroup analysis revealed distinct TBI risk patterns between HA and VHA regions. Occupational exposure was the predominant risk factor in HA areas, with manual laborers demonstrating a 3.5-fold increased risk. In contrast, VHA populations exhibited physiology-driven susceptibility, where advanced age (aOR=1.71), elevated BMI (aOR=1.35), and smoking (aOR=1.39) emerged as primary determinants. These epidemiological distinctions call for targeted interventions: workplace-focused measures, including ventilation standards and pre-employment screening in HA regions, versus prioritized screening of high-risk populations in VHA areas. The comorbidity patterns associated with TBI risk also differed significantly between HA and VHA regions. In HA areas, diabetes mellitus remained a significant risk factor for TBI (Lee et al., 2017), while VHA populations exhibited a distinct association between hypertension and TBI. Previous a systematic review indicated that there was a significant correlation between altitude and the prevalence of hypertension among inhabitants of Tibet, corresponding to a 2% increase in prevalence for every 100 m increase in altitude (Mingji et al., 2015). Moreover, in VHA regions, the respiratory system becomes particularly vulnerable (Talaminos-Barroso et al., 2021), where smoking demonstrates an altitude-dependent increase in TBI risk, while exercise may paradoxically diminish its protective immune benefits, likely due to compounded respiratory stress under hypoxic conditions. Therefore, these findings advocate for altitude-adapted comorbidity screening protocols, particularly emphasizing rigorous cardiopulmonary monitoring in VHA regions.

This study also has some limitations. First, because many men and young individuals were not at home during the survey period, there was a gap between our research population and the general population in Tibet. Although we adjusted the population structure based on the model, the estimated prevalence may still be biased (Floyd et al., 2013). After statistical adjustments, best-practice analytical methods estimated the prevalence of TBI among people aged 5 years or older in China to be 20.5% (95% CI 14.2%- 32.8%), which was at least 30% higher than the models without adjustment. The gap may be due to the difference between the sampled population structure and the general population structure and sampling. The proportion of female participants (55%) was higher than that in the general population (49%). In addition, simple random sampling increases the probability of communities or villages with low population density being selected, which may reduce the crude rate. Second, the WHO recommended the EC skin test, a newer MTB antigen-based skin test as an alternative to diagnose TBI, in 2022. However, it has not been extensively validated in people living in plateau areas. Although randomized diagnostic trials have shown that this positive determination criterion demonstrated high specificity and sensitivity of cutoff of ≥5 mm redness or induration, it might not be the most appropriate cutoff for this study population.

Our findings demonstrate that while the overall prevalence of TBI in Tibet (20.5%) is comparable to China’s national average, the region exhibits critical altitude-specific epidemiological characteristics that demand focused attention. These findings highlight the need to increase investment in marginalized populations, particularly high-altitude communities that face complex environmental and physiological challenges, to identify unique barriers to tuberculosis elimination and develop targeted tuberculosis control strategies.

## Data Availability

The raw data supporting the conclusions of this article will be made available by the authors, without undue reservation.
