# Supplementary material for: Epidemiological features of tuberculosis infection in a high-altitude population: a population-based, cross-sectional survey in Tibet, China
Source: Front Cell Infect Microbiol. 2025 Sep 2;15:1651920. doi: 10.3389/fcimb.2025.1651920 (PMC12436351; doi:10.3389/fcimb.2025.1651920)
Supplement: Supplementary file 1 [file Table1.docx]

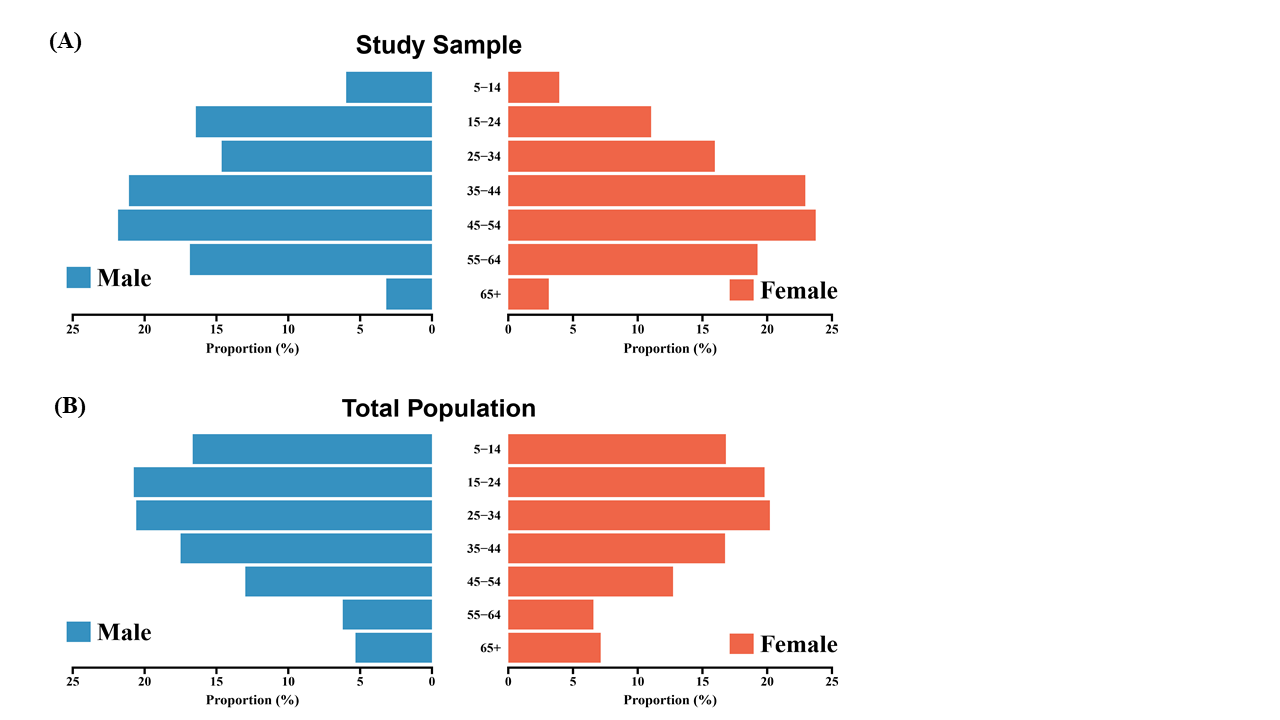


Figure S1: Age and sex distribution of (A) the participating population in our survey and (B) the general population of Tibet


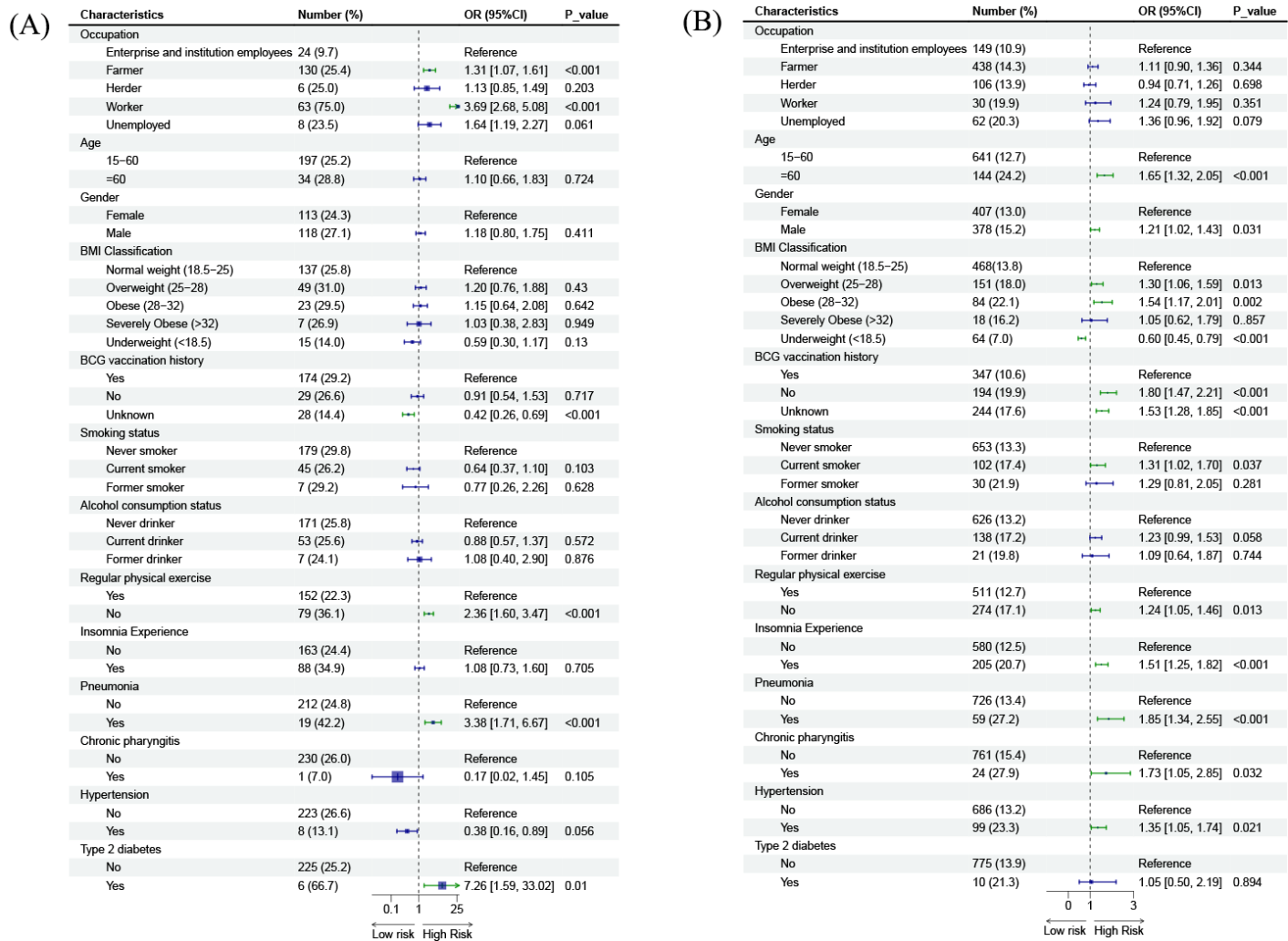


Figure S2: Multivariable Analysis of EC Skin Test Positivity Risk Factors at high and very high-altitude regions: (A) high altitude region; (B) very high-altitude region.


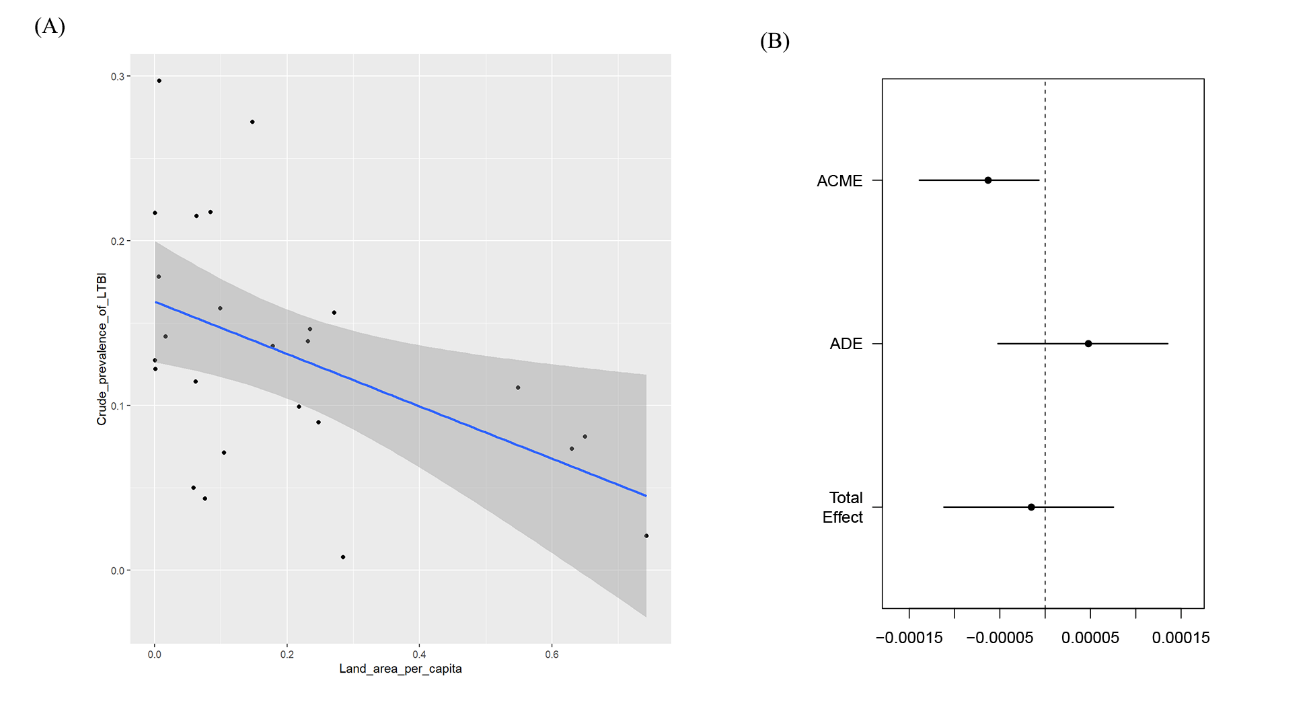


Figure S3: (A) Linear regression between crude prevalence of LTBI and Land area per capita; (B) Mediation Effect of Per Capita Living Area on the Altitude-LTBI Prevalence Relationship
