# Supplementary material for: Epidemiological features of tuberculosis infection in a high-altitude population: a population-based, cross-sectional survey in Tibet, China
Source: Front Cell Infect Microbiol. 2025 Sep 2;15:1651920. doi: 10.3389/fcimb.2025.1651920 (PMC12436351; doi:10.3389/fcimb.2025.1651920)
Supplement: Supplementary file 2 [file Table2.docx]

**I. Basic Information**

1. Gender: (1) Male (2) Female

2. Nationality: (1) Tibetan (2) Hui (3) Han (4) Other

3. ID card number:

4. Height: _ _ _._ _cm

5. Weight: _ _._ _kg

6. Relationship between you and the interviewee: (1) Yourself (2) Family member/Guardian (3) Other

7. How long have you lived in the local area?

(1) <6 months (2) 6 months to 3 years (3) 3-5 years (4) More than 5 years

8. Will you leave your place of residence in the next year? (1) Yes (2) No

9. Your marital status: (1) Single (2) Married or cohabiting (3) Divorced or separated (4) Widowed

10. Your educational level: (1) Illiterate (2) Primary school (3) Junior high school (4) Technical secondary school/high school (5) College (6) Undergraduate (7) Postgraduate and above

11. Your occupation: (1) Government agency, enterprise or institution (2) Commercial/service industry employee (3) Farmer (4) Herder (5) Student (6) Migrant worker (7) Retired person (8) Domestic worker (9) Worker (10) Unemployed

12. How do you interact with cattle, sheep and other livestock (multiple choices)?

(1) Milking (2) Shearing (3) Feeding (4) Grazing (5) Slaughtering (6) No contact

13. Do humans and animals live together: (1) Yes (2) No

14. Is the house ventilated regularly: (1) Yes (2) No

15. Altitude of residence: ___meters.

16. Have you ever been vaccinated with BCG: (1) Yes (2) No (3) Can't remember, don't know

**II Lifestyle**

17. Do you smoke? (1) Yes (2) No (3) Have quit smoking (at least quit smoking for more than 3 months)

18. Do you drink alcohol? (1) Yes (2) No (3) Have quit drinking (at least quit drinking for more than 1 year)

19. Do you exercise regularly? (1) Yes (2) No (jump to question 24 of this section)

20. Have you ever had insomnia? (1) Almost never (2) < 1 time/week (3) 1-2 times/week (4) > 3 times/week

21. Do you have a regular schedule? (1) Yes (2) No

**III History of disease**

22. Have you ever been diagnosed with the following diseases? (Multiple choices are allowed) (1) Undiagnosed diseases (2) Asthma (3) Pneumonia (4) Chronic pharyngitis (5) Chronic bronchitis (6) Chronic rhinitis (7) Essential hypertension (8) Cancer (9) Hepatitis (10) Diabetes mellitus II (11) Heart disease (12) Stroke

**IV History of Tuberculosis before this survey**

23. What is the type of pulmonary tuberculosis you have (please select one or more):

23.1 Have you ever been diagnosed with pulmonary tuberculosis;

(1) Yes; Diagnosis date: ____, (2) No

Treatment status: □ Cured; □ Stopped treatment; □ Treated; □ Untreated;

23.2 Have you ever been diagnosed with Tuberculous pleurisy;

(1) Yes; Diagnosis date: ____, (2) No

Treatment status: □ Cured; □ Stopped treatment; □ Treated; □ Untreated;

23.3 Have you ever been diagnosed with Extrapulmonary tuberculosis;

(1) Yes; Diagnosis date: ____, (2) No

Treatment status: □ Cured; □ Stopped treatment; □ Treated; □ Untreated;

23.4 Have you ever been diagnosed with Types of extrapulmonary tuberculosis:

(1) Yes; Diagnosis date: ____, (2) No

□ Cervical lymph node tuberculosis; □ Intestinal tuberculosis; □ Bone tuberculosis; □ Renal tuberculosis; □ Nervous system tuberculosis; □ Tuberculous meningitis; □ Other

**V symptom**

24. Did you experience any symptoms in the two weeks before this survey?

(1) Yes (2) No

25. If yes, which of the following symptoms did you experience (multiple choices are allowed)

(1) Coughing and sputum production (2) Hemoptysis or blood-tinged sputum (3) Chest pain (4) Shortness of breath (5) Fever (6) Night sweats (7) Fatigue (8) Unexplained significant weight loss (9) Decreased activity level
